# Accountable care organization changes in equity of ambulatory care quality by patient race and ethnicity, 2019-2022

**DOI:** 10.1093/haschl/qxae155

**Published:** 2024-11-21

**Authors:** Hector P Rodriguez, Shihan Xu, Amanda L Brewster, Timothy T Brown, Stacy Chen, Sarah D Epstein

**Affiliations:** Division of Health Policy and Management, School of Public Health, University of California, Berkeley, 94704, United States of America; Division of Health Policy and Management, School of Public Health, University of California, Berkeley, 94704, United States of America; Division of Health Policy and Management, School of Public Health, University of California, Berkeley, 94704, United States of America; Division of Health Policy and Management, School of Public Health, University of California, Berkeley, 94704, United States of America; Division of Health Policy and Management, School of Public Health, University of California, Berkeley, 94704, United States of America; Division of Health Policy and Management, School of Public Health, University of California, Berkeley, 94704, United States of America

**Keywords:** accountable care organizations, health equity, health disparities, value-based payment, payment reform

## Abstract

There is limited information about accountable care organization (ACO) variation in equity of ambulatory care quality. We examine whether equity of care changed for racial and ethnic minority patients from 2019 to 2022 and the extent to which equity of care performance varied for 11 ACOs in Massachusetts over time. We analyzed ACO-level changes in equity of care for 8 ambulatory care quality measures for Asian, Black, and Hispanic patients, measured as the percentage point difference between each group and the majority non-Hispanic White patient group. Cervical cancer screening (3.54 percentage point change, *P* < 0.001), colorectal cancer screening (3.54 percentage point change, *P* < 0.001), and eye exams for adults with diabetes (3.56 percentage point change, *P* = 0.008) had the largest performance declines. Equity of ambulatory care quality did not significantly change over time. The one exception was for breast cancer screening, where equity declined for Asian patients (3.52 percentage point change, *P* = 0.04). Although equity of care generally did not significantly change over time across ACOs, high variation in equity of care performance between ACOs highlights opportunities to identify and share the strategies that enable physician practices and healthcare systems to advance equity of care for racial and ethnic minority patients.

## Teaser

Despite increased policy attention focused on improving equity of care for racial and ethnic minority patients, there is limited information about organization-level variation in equity of ambulatory care quality. This study examines whether equity of ambulatory care quality changed for racial and ethnic minority patients from 2019 to 2022 across 11 accountable care organizations (ACOs) in Massachusetts. Overall quality declined for 5 of the 8 quality measures, but equity of care generally did not change significantly over time. Equity of care, however, varied widely between ACOs, highlighting opportunities to identify and share the strategies that enable physician practices and healthcare systems to advance equity of care for racial and ethnic minority patients.

## Introduction

Racial and ethnic minority patients experience more barriers to accessing healthcare and are less likely to receive evidence-based preventive and chronic care compared to non-Hispanic White patients.^1,2^ The healthcare systems, hospitals, and physician organizations that make up accountable care organizations (ACOs) vary in their capacity to address racial inequities in ambulatory care quality,^3^ with some organizations making new investments to address racial and ethnic inequities in care.

In spite of increased policy attention focused on improving equity of care,^4-6^ there is limited information about how ACOs vary in their provision of equitable care for racial and ethnic minority populations and how equity of care changes over time.^7^ Beginning in March 2022, Blue Cross Blue Shield of Massachusetts (BCBSMA) launched an equity improvement initiative to encourage ACOs to test equity-focused interventions and provided grant support to aid with improving their data and quality improvement infrastructure to improve racial equity of care.^8^ The BCBSMA and ACOs also negotiated 2023 and 2024 contracts to include financial incentives for equity of care based on the ambulatory care quality measures.^9^ We examine how 11 of the ACOs contracted with BCBSMA varied in their ability to advance equity of Healthcare Effectiveness Data and Information Set (HEDIS) ambulatory care quality by patient race and ethnicity prior to implementing performance-based financial incentives for equity of care.

## Methods

### Data

We analyzed healthcare claims and intermediate clinical outcomes data (2019-2022) from BCBSMA that included adult (*n* = 2 705 979 patient-years) and pediatric (*n* = 728 720 patient-years) patients across 11 ACOs participating in BCBSMA's equity improvement initiative. The data are restricted to BCBSMA members who qualify for 1 or more of the HEDIS ambulatory care quality measures included in the study.

Nine of the ACOs are led by hospital organizations and/or healthcare systems, and 2 ACOs are led by physician organizations. The ACOs all participate in BCBSMA's alternative quality contract (AQC), which is a 2-sided contract with shared savings if spending is below budget and shared risk if spending exceeds the budget. The ACOs receive quality bonuses that are based on quality of care and patients' experiences in the ambulatory care and hospital settings.^10^

### Measures

We conceptualize equity of care based on the National Academy of Medicine's definition of equitable care as “providing care that does not vary in quality because of personal characteristics such as gender, ethnicity, geographic location, and socioeconomic status”.^11^ Equity of care was measured as the difference in quality for the ethnic and racial minority patient group compared to the non-Hispanic White patient group. Non-Hispanic White patients were the largest patient group across ACOs and quality measures.

Eight HEDIS ambulatory care quality measures for each ACO were examined: (1) breast cancer screening, (2) cervical cancer screening, (3) colorectal cancer screening, (4) retinal eye exam, (5) poor HbA1c control (>9.0%) for adults with diabetes, (6) blood pressure control (<140/90 mm Hg) for adults with diabetes, (7) blood pressure control for adults with hypertension, and (8) annual well child and adolescent visits (Table S1 summarizes measure definitions). Given shelter-in-place ordinances and limited options for blood pressure and HbA1c testing, blood pressure control and hypertension measures were modified to allow 2019 measurements for eligible patients to count for 2020 performance. No other modifications to measure definitions were made over time.

The ACOs with less than 50 annual eligible patients for any one of the racial and ethnic minority patient groups for a given quality measure were excluded from the analyses of the measure due to concerns about unreliable estimation of percent denominator changes with small samples. As a result, analyses of “Diabetes: BP control”, “Diabetes: HbA1c poor control (>9.0%),” and “Diabetes: Eye Exam (Retinal) Performed” are applicable to 8 of the 11 ACOs; measurements of “Breast Cancer Screening” and “Child and Adolescent Well-Care Visits” are applicable to 10 of the 11 ACOs.

Patient race and ethnicity data included 4 patient groups: Asian, Black, Hispanic, and non-Hispanic White. These data included a combination of self-reported data (∼26%) and imputed data (∼74%) using the modified Bayesian Improved First Name Surname and Geocoding method,^12^ which uses surnames, first names, and residential addresses to indirectly estimate race and ethnicity for individuals missing self-reported data.

### Analysis

We conducted 2 sets of regression analyses: one focused on quality of care changes over time and one focused on equity of care changes over time. The unit of analysis was the ACO-year (44 ACO-level observations) for each of the 8 HEDIS measures. To examine whether temporal changes in quality of care were statistically significant (*P* < 0.05) for each racial and ethnic group, we estimated linear regression models that included continuous year (2019-2022) as a covariate for each of the 8 quality measures. Other research studies with modest samples have used similar methods.^13^

Then, we examined ACO variation in equity of care over time for each of the 8 HEDIS quality measures and for each racial/ethnic minority patient group compared to non-Hispanic White patients. To do this, we separately estimated linear regression models for each of the 8 equity of care measures (compared to non-Hispanic Whites) for each racial and ethnic minority group that included continuous year as a covariate.

Finally, we examined patient denominators for each measure over time by patient race and ethnicity because large denominator changes may complicate ACO efforts to monitor and improve equity of care.^9^

The study was approved by the [redacted] Institutional Review Board.

## Results

**Figure 1.**
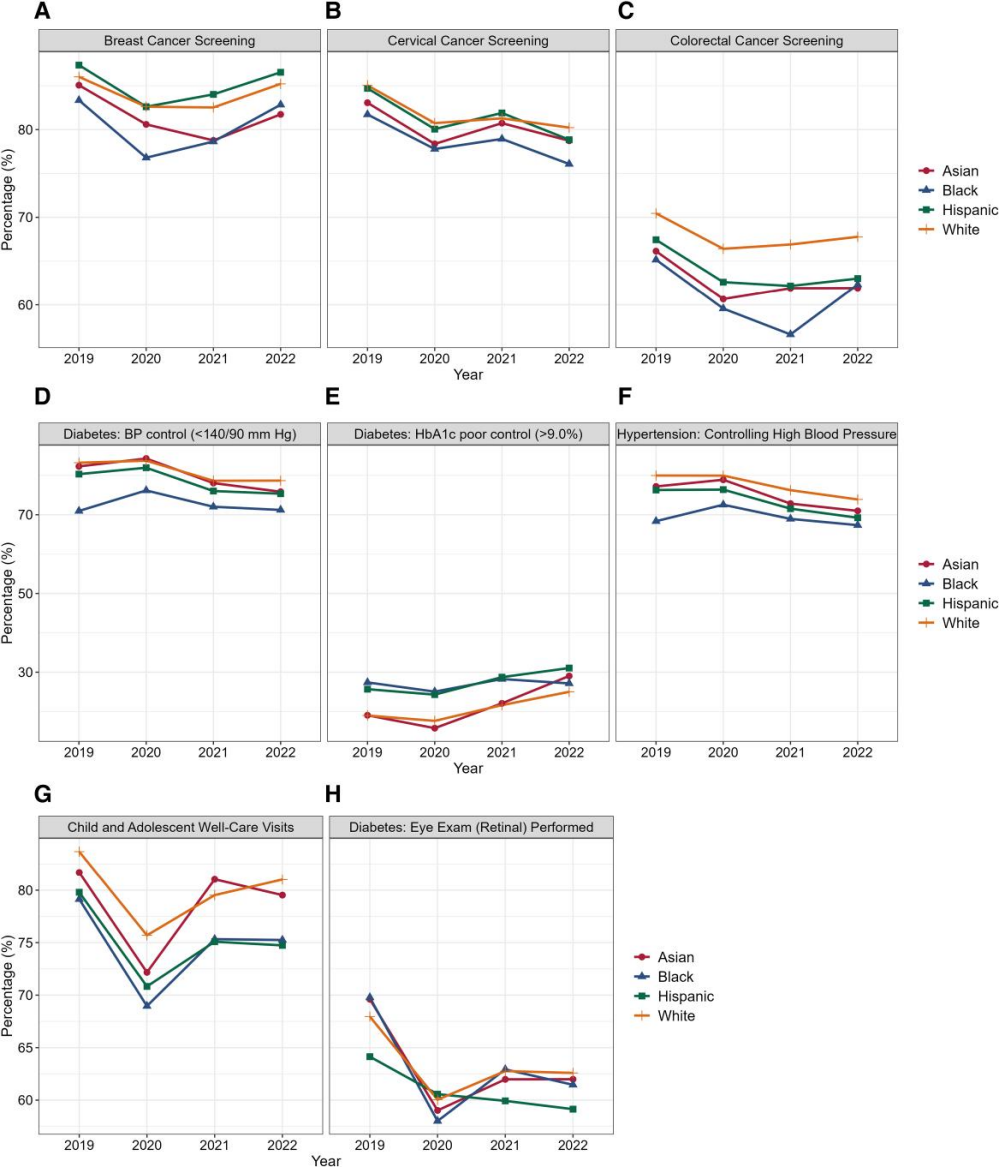
Changes in quality of ambulatory care by patient race and ethnicity, 2019-2022. Based on minimum denominators required for each racial and ethnic group (*n* = 50 per year), measurements of “Diabetes: BP control” (D), “Diabetes: HbA1c poor control (>9.0%)”, (E) and “Diabetes: Eye Exam (Retinal) Performed” (H) are applicable to 8 ACOs; measurements of “Breast Cancer Screening” (A) and “Child and Adolescent Well-Care Visits” (G) are applicable to 10 ACOs.

From 2019 to 2022, overall quality decreased for 5 of the 8 HEDIS measures across the 11 ACOs. Measures with the largest overall declines were cervical cancer screening (3.54 percentage point change, *P* < 0.001), colorectal cancer screening (3.54 percentage point change, *P* < 0.001), and eye exams for adults with diabetes (3.56 percentage point change, *P* = 0.008) (Figure 1; Table S2 reports annual estimates).

**Figure 2.**
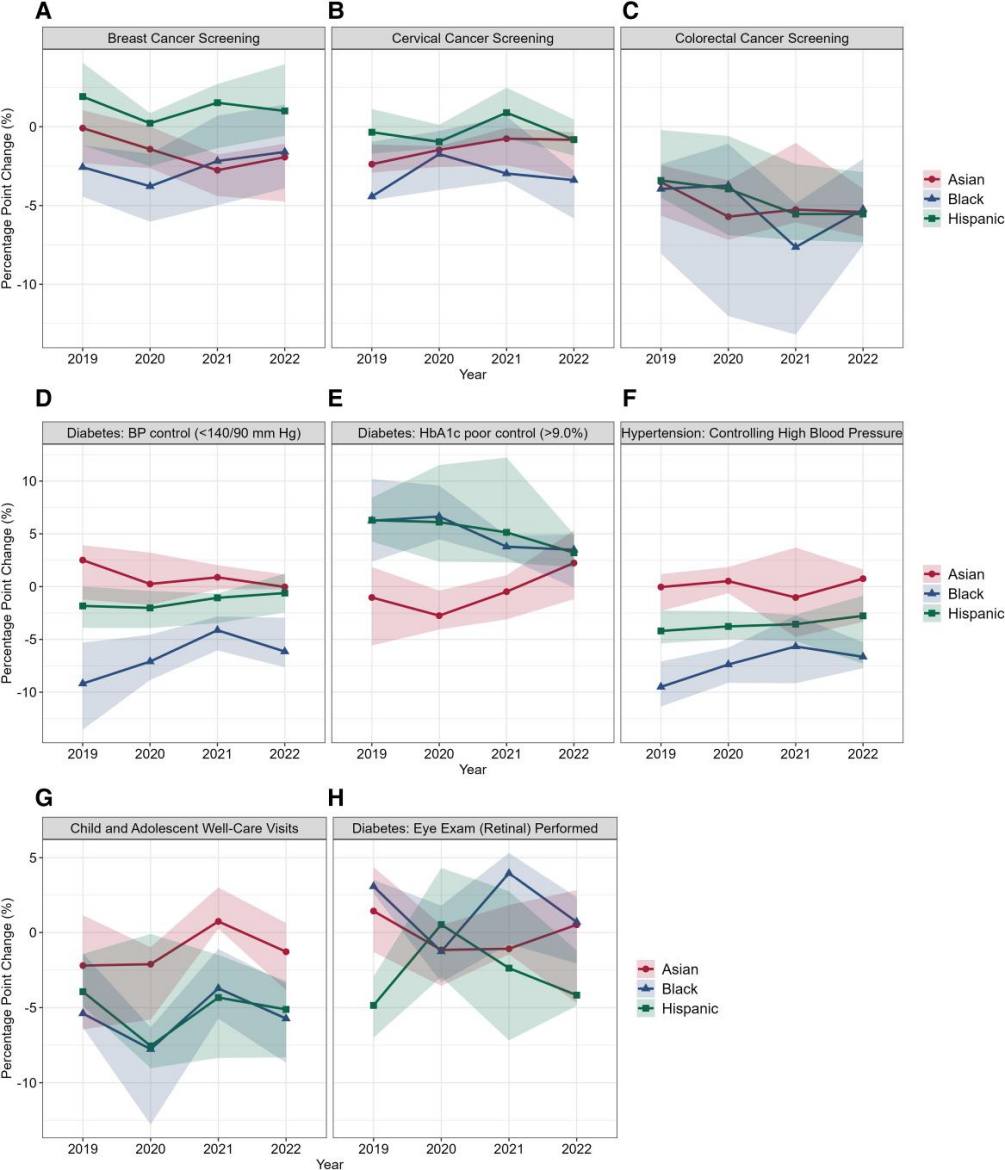
Variation in accountable care organization changes in equity of care, by ambulatory care quality measure and patient race and ethnicity, 2019-2022. Equity of care for each racial/ethnic minority patient group was calculated as the difference relative to non-Hispanic White patients as the reference group, which was the largest group across ACOs and measures. Based on minimum denominators required for each racial/ethnic group (minimum of 50 per year), measurements of “Diabetes: BP control” (D), “Diabetes: HbA1c poor control (>9.0%)” (E), and “Diabetes: Eye Exam (Retinal) Performed” (F) are applicable to 8 ACOs; measurements of “Breast Cancer Screening” (A) and “Child and Adolescent Well-Care Visits” (G) are applicable to 10 ACOs.

Of the 8 HEDIS ambulatory care measures, only colorectal cancer screening for Asian patients had a statistically significant decline in equity over time compared to non-Hispanic White patients. Asian patients had a 3.52 percentage point (*P* = 0.04) decline in equity of care relative to non-Hispanic White patients (Figure 2; Table S3 reports annual estimates).

Equity of care for the 8 HEDIS ambulatory care quality measures, however, varied widely between the 11 ACOs over time (Figure 2). Colorectal cancer screening had the largest ACO variation in equity of care (Figure 2C). For example, in 2020, equity of colorectal cancer screening for Black patients was −3.7 percentage points for the median ACO in 2020, −1.09 percentage points for the ACO at the 75th percentile, and −12.03 percentage points for the ACO at the 25th percentile of equity performance distribution for Black patients (Figure 2C).

Percent changes in patient denominators between 2019 and 2022 were larger for Hispanic and Asian patients than for non-Hispanic White patients (Table S4). Total patient counts by ACO per measure and year are detailed in Table S5.

## Discussion

Quality of care declined from 2019 to 2022 for 5 of the 8 HEDIS ambulatory care quality measures, but equity of care generally did not significantly change over time for commercially insured patients of 11 ACOs operating in Massachusetts. We found high variation in equity of care performance between the ACOs over time, indicating that some physician practices and healthcare systems of ACOs continued to maintain equity of care for racial and ethnic minority patients despite declines in global quality, whereas some organizations struggled to maintain equity of care. There may be opportunities to advance equity by identifying and sharing effective strategies used by the ACOs to maintain and improve equity of care by patient race and ethnicity.

The differential increases in patient denominators by patient race and ethnicity highlight a potential challenge for equity initiatives because new patients may be less likely to have quality of care measures met compared to established patients.^14^ Past evidence indicates that insurance instability can impact quality of care among racial and ethnic minority patients,^15^ so the impact of insurance instability on equity of care performance should be monitored as equity measures and incentives are integrated into value-based payment arrangements.

The study's limitations should be considered. First, the generalizability of the findings could be limited by the Massachusetts context and due to BCBSMA's market share, which enabled reliable analyses of equity of care by patient race and ethnicity. Second, data prior to 2019 and after 2022 are not available, so additional years could not be examined. Third, analyses of equity of care over time by patient race and ethnicity had limited statistical power, and only large changes in equity of care could be identified at the *P* < 0.05 significance level. This may be one reason why overall quality of care declined consistently, while equity of care did not. Finally, patient demographic and clinical characteristics were not examined to understand the role of ACOs' patient case mix on changes in equity of care. When national samples of ACO-level equity of care data are available, these relationships should be rigorously examined.

As performance-based financial incentives to improve equity of care by patient race and ethnicity are implemented by payers, the high variation in equity of care between ACOs highlights opportunities to identify and share the strategies that enable ACOs to advance equity of ambulatory care quality for racial and ethnic minority patients. More evidence is needed to understand the impact of equity initiatives being implemented by healthcare organizations and how these efforts impact equity of care by patient race and ethnicity.

## Supplementary Material

qxae155_Supplementary_Data
