# Supplementary material for: Accountable care organization changes in equity of ambulatory care quality by patient race and ethnicity, 2019-2022
Source: Health Aff Sch. 2024 Nov 21;2(12):qxae155. doi: 10.1093/haschl/qxae155 (PMC11632828; doi:10.1093/haschl/qxae155)
Supplement: qxae155_Supplementary_Data [file qxae155_supplementary_data.zip › Supp_Changes_EquityofCare_ACOs2.docx]

**Accountable Care Organization Changes in Equity of Ambulatory Care Quality**

**by Patient Race and Ethnicity, 2019-2022**

Online Material Table of Contents

**Online Table 1:** Healthcare Effectiveness Data and Information Set (HEDIS) Measure Specifications

**Online Table 2:** Regression Model Results: Changes in Ambulatory Care Quality (2019 to 2022), by Measure and by Patient Race and Ethnicity

**Online Table 3:** Regression Model Results: Changes in Equity of Ambulatory Care Quality by Race and Ethnicity, 2019-2022

**Online Table 4:** Percent Denominator Change Over Time for Ambulatory Care Quality Measures (2019 to 2022), by Accountable Care Organization and Patient Race and Ethnicity

**Online Table 5:** Denominators for Ambulatory Care Quality Measures (2019 to 2022), by Accountable Care Organization and Patient Race and Ethnicity

**Online Table 1: Healthcare Effectiveness Data and Information Set (HEDIS) Measure Specifications**

Equity of care is measured as the difference in percentage points between the racial/ethnic minority patient group and non-Hispanic White patients for each of the 8 quality of care measures.

**1.** **Breast Cancer Screening**

% of women who had at least one mammogram within the past 2 years, ages 50-74

**2.** **Cervical Cancer Screening**

% of women who were screened for cervical cancer using appropriate guidelines, ages 21-64

Methods:

- Members 21–64 years of age who were recommended for routine cervical cancer screening and had cervical cytology performed within the last 3 years.
- Members 30–64 years of age who were recommended for routine cervical cancer screening and had cervical high-risk human papillomavirus (hrHPV) testing performed within the last 5 years.
- Members 30–64 years of age who were recommended for routine cervical cancer screening and had cervical cytology/high-risk human papillomavirus (hrHPV) cotesting within the last 5 years.

**3.** **Colorectal Cancer Screening**

% of adult members who had appropriate screening for colorectal cancer, ages 50-75

Methods:

- Annual fecal occult blood test
- Flexible sigmoidoscopy every 5 years
- Colonoscopy every 10 years
- Computed tomography colonography every 5 years
- Stool DNA test every 3 years.

**4.** **Comprehensive Diabetes Care - Retinal Eye Exam**

% of adult diabetic members who had eye exams performed, ages 18-75

**5.** **Comprehensive Diabetes Care - HbA1c Poor Control (lower rates indicate higher quality care)**

% of adult diabetic members with uncontrolled HbA1c, defined as HbA1c > 9.0, ages 18-75

**6.** **Comprehensive Diabetes Care – Blood Pressure Control**

% of adult diabetic members with blood pressure controlled, defined as (<140/90 mm Hg), ages 18-74

**7.** **Controlling High Blood Pressure**

% of adult hypertensive members who keep their blood pressure controlled, defined as (<140/90 mm Hg), ages 18-85

**8.** **Child and Adolescent Well-Care Visits**

% of child and adolescent members who had at least one comprehensive well-care visit with a PCP or OB/GYN, ages 3-21.

**Online Table 2: Regression Model Results: Changes in Ambulatory Care Quality (2019 to 2022), by Measure and by Patient Race and Ethnicity**

| **Quality Measure** | **Race / Ethnicity** | **Percentage point change per year** | **P-value** |
| --- | --- | --- | --- |
| **Breast Cancer Screening** | Overall | 0.467 | 0.16 |
|  | Asian | 0.894 | 0.02* |
|  | Black | 0.043 | 1 |
|  | Hispanic | 0.336 | 0.36 |
|  | White | 0.338 | 0.32 |

| **Cervical Cancer Screening** | Overall | 0.885 | p<0.001*** |
| --- | --- | --- | --- |
|  | Asian | 0.652 | 0.05 |
|  | Black | 0.810 | 0.01* |
|  | Hispanic | 0.885 | p<0.001*** |
|  | White | 0.885 | p<0.001*** |

| **Colorectal Cancer Screening** | Overall | 0.885 | p<0.001*** |
| --- | --- | --- | --- |
|  | Asian | 0.770 | 0.02* |
|  | Black | 0.731 | 0.03* |
|  | Hispanic | 0.804 | 0.005** |
|  | White | 0.885 | p<0.001*** |

| **Diabetes: BP control (<140/90 mm Hg)** | Overall | 0.545 | 0.15 |
| --- | --- | --- | --- |
|  | Asian | 0.575 | 0.16 |
|  | Black | 0.128 | 0.84 |
|  | Hispanic | 0.297 | 0.46 |
|  | White | 0.594 | 0.11 |

| **Diabetes: HbA1c poor control (>9.0%)** | Overall | 0.198 | 0.64 |
| --- | --- | --- | --- |
|  | Asian | 0.575 | 0.16 |
|  | Black | 0.128 | 0.84 |
|  | Hispanic | 0.297 | 0.46 |
|  | White | 0.396 | 0.31 |

| **Hypertension: Controlling High Blood Pressure** | Overall | 0.751 | p<0.001*** |
| --- | --- | --- | --- |
|  | Asian | 0.639 | 0.11 |
|  | Black | 0 | 1 |
|  | Hispanic | 0.532 | 0.11 |
|  | White | 0.751 | p<0.001*** |

| **Child and Adolescent Well-Care Visits** | Overall | 0.854 | 0.004** |
| --- | --- | --- | --- |
|  | Asian | 0.533 | 0.13 |
|  | Black | 0.770 | 0.02** |
|  | Hispanic | 0.822 | 0.006** |
|  | White | 0.854 | 0.004** |

| **Diabetes: Eye Exam (Retinal) Performed** | Overall | 0.891 | 0.008** |
| --- | --- | --- | --- |
|  | Asian | 0.831 | 0.03* |
|  | Black | 0.899 | 0.03* |
|  | Hispanic | 0.891 | 0.008** |
|  | White | 0.842 | 0.016* |

Noe: * p<0.05, ** p<0.01, ***p<0.001

**Online Table 3: Regression Model Results: Changes in Equity of Ambulatory Care Quality by Race and Ethnicity, 2019-2022**

| **Quality Measure** | **Patient Race and Ethnicity** | **Model Term** | **Percentage point change per year** | **P-value** | **Model R-squared** |
| --- | --- | --- | --- | --- | --- |
| **Breast Cancer**  **Screening** | Asian | (Intercept) | 1775.86 | 0.04* | 0.16 |
|  |  | Year | -0.88 | 0.04* |  |
|  | Black | (Intercept) | -793.63 | 0.66 | 0.01 |
|  |  | Year | 0.39 | 0.66 |  |
|  | Hispanic | (Intercept) | -412.37 | 0.76 | 0.00 |
|  |  | Year | 0.20 | 0.76 |  |
| **Cervical Cancer**  **Screening** | Asian | (Intercept) | -499.53 | 0.57 | 0.01 |
|  |  | Year | 0.25 | 0.57 |  |
|  | Black | (Intercept) | 544.82 | 0.60 | 0.01 |
|  |  | Year | -0.27 | 0.60 |  |
|  | Hispanic | (Intercept) | 371.32 | 0.63 | 0.01 |
|  |  | Year | -0.18 | 0.63 |  |
| **Child and Adolescent Well-Care Visits** | Asian | (Intercept) | -1470.86 | 0.32 | 0.03 |
|  |  | Year | 0.73 | 0.32 |  |
|  | Black | (Intercept) | 354.56 | 0.85 | 0.00 |
|  |  | Year | -0.18 | 0.85 |  |
|  | Hispanic | (Intercept) | 1325.91 | 0.38 | 0.02 |
|  |  | Year | -0.66 | 0.38 |  |
| **Colorectal Cancer**  **Screening** | Asian | (Intercept) | 568.58 | 0.52 | 0.01 |
|  |  | Year | -0.28 | 0.51 |  |
|  | Black | (Intercept) | 567.51 | 0.78 | 0.00 |
|  |  | Year | -0.28 | 0.78 |  |
|  | Hispanic | (Intercept) | 1258.73 | 0.21 | 0.04 |
|  |  | Year | -0.63 | 0.21 |  |
| **Diabetes: BP control**  **(<140/90 mm Hg)** | Asian | (Intercept) | 2094.02 | 0.22 | 0.06 |
|  |  | Year | -1.04 | 0.22 |  |
|  | Black | (Intercept) | -2493.10 | 0.15 | 0.09 |
|  |  | Year | 1.23 | 0.15 |  |
|  | Hispanic | (Intercept) | 1033.47 | 0.53 | 0.01 |
|  |  | Year | -0.51 | 0.53 |  |
| **Diabetes: Eye Exam (Retinal) Performed** | Asian | (Intercept) | 1781.07 | 0.43 | 0.02 |
|  |  | Year | -0.88 | 0.43 |  |
|  | Black | (Intercept) | 1399.24 | 0.37 | 0.04 |
|  |  | Year | -0.69 | 0.38 |  |
|  | Hispanic | (Intercept) | 338.29 | 0.83 | 0.00 |
|  |  | Year | -0.17 | 0.83 |  |
| **Diabetes: HbA1c**  **poor control (>9.0%)** | Asian | (Intercept) | -2913.94 | 0.06 | 0.13 |
|  |  | Year | 1.44 | 0.06 |  |
|  | Black | (Intercept) | 2441.23 | 0.16 | 0.09 |
|  |  | Year | -1.21 | 0.16 |  |
|  | Hispanic | (Intercept) | -255.44 | 0.90 | 0.00 |
|  |  | Year | 0.13 | 0.90 |  |
| **Hypertension:**  **Controlling High**  **Blood Pressure** | Asian | (Intercept) | 998.89 | 0.51 | 0.02 |
|  |  | Year | -0.49 | 0.51 |  |
|  | Black | (Intercept) | -1998.50 | 0.12 | 0.08 |
|  |  | Year | 0.99 | 0.13 |  |
|  | Hispanic | (Intercept) | 884.78 | 0.55 | 0.01 |
|  |  | Year | -0.44 | 0.54 |  |

**Online Table 4: Percent Denominator Change Over Time for Ambulatory Care Quality Measures (2019 to 2022), by Accountable Care Organization and Patient Race and Ethnicity**

Breast Cancer Screening

| **ACO Number** | **Overall** | **Asian** | **Black** | **Hispanic** | **White** |
| --- | --- | --- | --- | --- | --- |
| 1 | 10% | 40% | 35% | 41% | 7% |
| 2 | 26% | 47% | 21% | 46% | 19% |
| 3 | 57% |  |  | 108% | 55% |
| 4 | -1% | 40% | 9% | 4% | -3% |
| 5 | 19% | 55% | 13% | 25% | 18% |
| 6 | 6% | 28% |  | 8% | 6% |
| 7 | 10% |  |  |  | 10% |
| 8 | -8% |  |  | -15% | -8% |
| 9 | 28% | 99% | 46% | 28% | 27% |
| 10 |  |  |  |  |  |
| 11 | 7% | 28% | -2% | 14% | 6% |
| Overall | 12% | 45% | 19% | 23% | 11% |

Cervical Cancer Screening

| **ACO Number** | **Overall** | **Asian** | **Black** | **Hispanic** | **White** |
| --- | --- | --- | --- | --- | --- |
| 1 | 12% | 31% | 32% | 38% | 8% |
| 2 | 22% | 39% | 21% | 20% | 21% |
| 3 | 65% | 81% | 256% | 141% | 60% |
| 4 | -2% | 21% | 6% | -2% | -5% |
| 5 | 22% | 48% | 31% | 25% | 21% |
| 6 | 1% | 5% | 5% | 15% | 1% |
| 7 | 9% | 76% | 33% | 35% | 8% |
| 8 | -3% |  |  | 16% | -4% |
| 9 | 28% | 125% | 34% | 49% | 25% |
| 10 | -38% |  |  | -11% | -40% |
| 11 | 8% | 14% | -2% | 9% | 9% |
| Overall | 13% | 32% | 23% | 23% | 11% |

Child and Adolescent Well-Care Visits

| **ACO Number** | **Overall** | **Asian** | **Black** | **Hispanic** | **White** |
| --- | --- | --- | --- | --- | --- |
| 1 | 8% | 14% | 33% | 27% | 5% |
| 2 | -16% | -18% | -17% | -37% | 1% |
| 3 | -15% | -25% | -21% | -37% | -13% |
| 4 | -20% | -13% | -16% | -27% | -20% |
| 5 | 3% | 14% | 30% | -2% | 2% |
| 6 | 0% | 4% | 4% | -11% | -1% |
| 7 | -11% | 6% | -20% | -3% | -11% |
| 8 | -56% |  |  | -56% | -56% |
| 9 | -18% | -22% | -10% | -18% | -18% |
| 10 |  |  |  |  |  |
| 11 | -28% | -19% | -46% | -36% | -28% |
| Overall | -10% | 0% | -2% | -16% | -11% |

Colorectal Cancer Screening

| **ACO Number** | **Overall** | **Asian** | **Black** | **Hispanic** | **White** |
| --- | --- | --- | --- | --- | --- |
| 1 | 9% | 40% | 25% | 33% | 6% |
| 2 | 11% | 33% | 1% | 15% | 10% |
| 3 | 73% | 255% | 215% | 161% | 69% |
| 4 | -5% | 32% | 1% | -7% | -7% |
| 5 | 15% | 43% | 10% | 22% | 14% |
| 6 | 8% | 32% | 10% | 4% | 8% |
| 7 | 7% | 36% | 27% | 19% | 7% |
| 8 | -9% |  |  | -15% | -10% |
| 9 | 37% | 115% | 57% | 35% | 36% |
| 10 | -36% |  |  | -8% | -38% |
| 11 | 5% | 16% | -5% | 4% | 5% |
| Overall | 11% | 38% | 15% | 17% | 10% |

Diabetes: BP control (<140/90 mm Hg)

| **ACO Number** | **Overall** | **Asian** | **Black** | **Hispanic** | **White** |
| --- | --- | --- | --- | --- | --- |
| 1 | 33% | 38% | 98% | 69% | 22% |
| 2 | 11% | 30% | 0% | -11% | 31% |
| 3 | 35% |  |  | 71% | 30% |
| 4 | -3% | 17% | 7% | -15% | -4% |
| 5 | 15% | 36% | 36% | 26% | 12% |
| 6 | 8% | 16% |  | 10% | 6% |
| 7 |  |  |  |  |  |
| 8 |  |  |  |  |  |
| 9 | 12% | 77% | 55% | 9% | 10% |
| 10 |  |  |  |  |  |
| 11 | 1% | 12% | -7% | 0% | 1% |
| Overall | 4% | 27% | 32% | 10% | 0% |

Diabetes: Eye Exam

| **ACO Number** | **Overall** | **Asian** | **Black** | **Hispanic** | **White** |
| --- | --- | --- | --- | --- | --- |
| 1 | 33% | 38% | 98% | 69% | 22% |
| 2 | 11% | 30% | 0% | -11% | 31% |
| 3 | 35% |  |  | 71% | 30% |
| 4 | -3% | 17% | 7% | -15% | -4% |
| 5 | 15% | 36% | 36% | 26% | 12% |
| 6 | 8% | 16% |  | 10% | 6% |
| 7 |  |  |  |  |  |
| 8 |  |  |  |  |  |
| 9 | 12% | 77% | 55% | 9% | 10% |
| 10 |  |  |  |  |  |
| 11 | 1% | 12% | -7% | 0% | 1% |
| Overall | 2% | 27% | 32% | 13% | -1% |

Diabetes: HbA1c poor control (>9.0%)

| **ACO Number** | **Overall** | **Asian** | **Black** | **Hispanic** | **White** |
| --- | --- | --- | --- | --- | --- |
| 1 | 33% | 38% | 98% | 69% | 22% |
| 2 | 11% | 30% | 0% | -11% | 31% |
| 3 | 35% |  |  | 71% | 30% |
| 4 | -3% | 17% | 7% | -15% | -4% |
| 5 | 15% | 36% | 36% | 26% | 12% |
| 6 | 8% | 16% |  | 10% | 6% |
| 7 |  |  |  |  |  |
| 8 |  |  |  |  |  |
| 9 | 12% | 77% | 55% | 9% | 10% |
| 10 |  |  |  |  |  |
| 11 | 1% | 12% | -7% | 0% | 1% |
| Overall | 4% | 27% | 32% | 13% | 0% |

Controlling High Blood Pressure

| **ACO Number** | **Overall** | **Asian** | **Black** | **Hispanic** | **White** |
| --- | --- | --- | --- | --- | --- |
| 1 | -8% | 3% | 36% | 8% | -13% |
| 2 | -11% | 3% | -18% | -18% | -6% |
| 3 | 55% |  | 113% | 89% | 52% |
| 4 | -26% | -5% | -16% | -37% | -27% |
| 5 | -8% | 6% | -4% | -9% | -9% |
| 6 | -20% | -8% | -11% | -28% | -20% |
| 7 | 36% |  |  |  | 36% |
| 8 | -27% |  |  | -31% | -27% |
| 9 | 12% | 87% | 41% | 12% | 10% |
| 10 | -41% |  |  | -26% | -42% |
| 11 | -21% | -8% | -17% | -22% | -22% |
| Overall | -9% | 2% | 2% | -12% | -10% |

Note: The number of patients eligible to be included (i.e., denominators) for 6 of the 8 measures increased over time (range: 2-13%) with the exception of denominators for well visits for children and adolescents (-10%) and blood pressure control (-9%) Hispanic and Asian patients experienced relatively larger denominator increases than White patients; Asian patient denominators increased by more than 15% for 6 of 8 measures, while Hispanic patient denominators increased by more than 15% for 3 of 8 measures. Blank cells reflect measures for which the accountable care organization (ACO) did not meet the minimum denominator (50 patients) for the racial/ethnic minority group in one or both measurement years (2019 and 2022) for the measure.

**Online Table 5: Denominators for Ambulatory Care Quality Measures (2019 to 2022), by Accountable Care Organization and Patient Race and Ethnicity**

| **Breast Cancer Screening** | | | | | | | | | | | | | | | | | | | | |
| --- | --- | --- | --- | --- | --- | --- | --- | --- | --- | --- | --- | --- | --- | --- | --- | --- | --- | --- | --- | --- |
|  | **2019** | | | | | **2020** | | | | | **2021** | | | | | **2022** | | | | |
| Group | All | Asian | Black | Hispanic | White | All | Asian | Black | Hispanic | White | All | Asian | Black | Hispanic | White | All | Asian | Black | Hispanic | White |
| 1 | 10366 | 543 | 391 | 249 | 8996 | 10217 | 558 | 394 | 264 | 8782 | 9956 | 611 | 434 | 265 | 8489 | 11425 | 758 | 526 | 352 | 9620 |
| 2 | 1281 | 109 | 273 | 183 | 697 | 1483 | 114 | 300 | 212 | 837 | 1569 | 139 | 321 | 235 | 853 | 1610 | 160 | 331 | 268 | 829 |
| 3 | 3036 |  |  | 66 | 2919 | 2895 |  |  | 71 | 2777 | 2783 |  |  | 74 | 2663 | 4781 |  |  | 137 | 4528 |
| 4 | 19959 | 910 | 366 | 525 | 17936 | 19783 | 989 | 365 | 558 | 17583 | 19386 | 1117 | 377 | 500 | 17205 | 19761 | 1277 | 398 | 544 | 17354 |
| 5 | 24973 | 848 | 352 | 623 | 22824 | 40645 | 1369 | 532 | 1063 | 34967 | 28301 | 1090 | 365 | 719 | 25869 | 29782 | 1317 | 399 | 777 | 27027 |
| 6 | 3290 | 121 |  | 125 | 2977 | 3444 | 135 |  | 111 | 3123 | 3722 | 151 |  | 141 | 3366 | 3503 | 155 |  | 135 | 3158 |
| 7 | 3694 |  |  |  | 3531 | 3709 |  |  |  | 3511 | 4149 |  |  |  | 3945 | 4070 |  |  |  | 3874 |
| 8 | 1895 |  |  | 73 | 1806 | 1849 |  |  | 71 | 1761 | 1841 |  |  | 64 | 1758 | 1739 |  |  | 62 | 1654 |
| 9 | 9527 | 140 | 125 | 392 | 8765 | 11454 | 195 | 243 | 513 | 10396 | 14883 | 286 | 202 | 600 | 13686 | 12208 | 278 | 182 | 502 | 11172 |
| 10 | 876 |  |  |  | 816 | 751 |  |  |  | 704 | 615 |  |  |  | 579 |  |  |  |  |  |
| 11 | 9710 | 509 | 181 | 236 | 8619 | 9836 | 537 | 174 | 268 | 8700 | 10540 | 601 | 186 | 274 | 9357 | 10375 | 651 | 178 | 268 | 9162 |
| Overall | 88607 | 3180 | 1688 | 2472 | 79886 | 106066 | 3897 | 2008 | 3131 | 93141 | 97745 | 3995 | 1885 | 2872 | 87770 | 99254 | 4596 | 2014 | 3045 | 88378 |
| **Cervical Cancer Screening** | | | | | | | | | | | | | | | | | | | | |
|  | **2019** | | | | | **2020** | | | | | **2021** | | | | | **2022** | | | | |
| Group | All | Asian | Black | Hispanic | White | All | Asian | Black | Hispanic | White | All | Asian | Black | Hispanic | White | All | Asian | Black | Hispanic | White |
| 1 | 23700 | 2199 | 825 | 751 | 19467 | 23151 | 2218 | 794 | 768 | 18836 | 23277 | 2403 | 834 | 811 | 18852 | 26482 | 2890 | 1090 | 1039 | 21081 |
| 2 | 3346 | 388 | 547 | 545 | 1804 | 3759 | 424 | 591 | 600 | 2071 | 4053 | 483 | 663 | 674 | 2174 | 4088 | 538 | 663 | 652 | 2175 |
| 3 | 4495 | 47 | 32 | 137 | 4273 | 4386 | 38 | 29 | 158 | 4156 | 4390 | 40 | 33 | 183 | 4129 | 7395 | 85 | 114 | 330 | 6850 |
| 4 | 37360 | 3033 | 751 | 1373 | 31787 | 36669 | 3114 | 725 | 1338 | 30923 | 35635 | 3359 | 775 | 1268 | 29900 | 36466 | 3680 | 796 | 1340 | 30309 |
| 5 | 44653 | 2531 | 649 | 1582 | 39322 | 85786 | 4607 | 1428 | 3464 | 70373 | 50790 | 3158 | 771 | 1885 | 44485 | 54489 | 3740 | 853 | 1979 | 47411 |
| 6 | 7126 | 556 | 91 | 314 | 6111 | 7328 | 614 | 93 | 330 | 6216 | 7673 | 620 | 97 | 370 | 6556 | 7208 | 584 | 96 | 361 | 6144 |
| 7 | 7299 | 87 | 52 | 66 | 6856 | 7318 | 106 | 57 | 71 | 6815 | 8238 | 123 | 64 | 84 | 7710 | 7955 | 153 | 69 | 89 | 7411 |
| 8 | 3187 |  |  | 94 | 3039 | 3219 |  |  | 99 | 3077 | 3243 |  |  | 107 | 3088 | 3078 |  |  | 109 | 2918 |
| 9 | 16228 | 335 | 228 | 688 | 14735 | 20057 | 464 | 461 | 995 | 17891 | 25858 | 800 | 391 | 1199 | 23174 | 20716 | 755 | 306 | 1026 | 18464 |
| 10 | 1396 |  |  | 84 | 1266 | 1282 |  |  | 101 | 1143 | 1048 |  |  | 81 | 937 | 865 |  |  | 75 | 764 |
| 11 | 16615 | 1606 | 307 | 547 | 13911 | 16947 | 1655 | 297 | 587 | 14133 | 18032 | 1781 | 305 | 640 | 15122 | 17989 | 1828 | 302 | 597 | 15095 |
| Overall | 165405 | 10782 | 3482 | 6181 | 142571 | 209902 | 13240 | 4475 | 8511 | 175634 | 182237 | 12767 | 3933 | 7302 | 156127 | 186731 | 14253 | 4289 | 7597 | 158622 |
| **Child and Adolescent Well-Care Visits** | | | | | | | | | | | | | | | | | | | | |
|  | **2019** | | | | | **2020** | | | | | **2021** | | | | | **2022** | | | | |
| Group | All | Asian | Black | Hispanic | White | All | Asian | Black | Hispanic | White | All | Asian | Black | Hispanic | White | All | Asian | Black | Hispanic | White |
| 1 | 29610 | 3711 | 1158 | 1129 | 23236 | 32454 | 4162 | 1348 | 1343 | 25092 | 30938 | 3937 | 1253 | 1270 | 24127 | 31916 | 4244 | 1545 | 1434 | 24361 |
| 2 | 3440 | 507 | 902 | 923 | 1065 | 2972 | 395 | 697 | 792 | 1028 | 2819 | 406 | 654 | 717 | 988 | 2887 | 417 | 749 | 586 | 1079 |
| 3 | 4291 | 89 | 141 | 336 | 3710 | 1919 | 16 | 38 | 106 | 1755 | 3602 | 64 | 129 | 268 | 3133 | 3638 | 67 | 111 | 212 | 3237 |
| 4 | 18533 | 1692 | 521 | 946 | 15274 | 17682 | 1513 | 481 | 892 | 14631 | 16173 | 1482 | 424 | 822 | 13363 | 14810 | 1472 | 437 | 686 | 12157 |
| 5 | 48587 | 3475 | 934 | 2104 | 41532 | 50406 | 3700 | 1016 | 2134 | 42735 | 48919 | 3669 | 995 | 2029 | 41658 | 50105 | 3961 | 1216 | 2059 | 42310 |
| 6 | 11575 | 1480 | 236 | 609 | 9181 | 11752 | 1491 | 274 | 562 | 9307 | 11465 | 1490 | 249 | 581 | 9073 | 11521 | 1535 | 245 | 545 | 9128 |
| 7 | 12730 | 231 | 135 | 170 | 11913 | 13273 | 246 | 121 | 186 | 12403 | 12798 | 256 | 120 | 189 | 11962 | 11388 | 246 | 108 | 165 | 10616 |
| 8 | 1987 |  |  | 117 | 1818 | 1047 |  |  | 60 | 955 | 1701 |  |  | 94 | 1555 | 880 |  |  | 52 | 808 |
| 9 | 27104 | 757 | 539 | 1675 | 23957 | 15247 | 349 | 365 | 953 | 13477 | 25105 | 674 | 522 | 1608 | 22162 | 22182 | 593 | 486 | 1375 | 19603 |
| 10 | 1599 |  | 95 | 210 | 1250 | 713 |  | 27 | 77 | 591 | 1350 |  | 67 | 188 | 1068 |  |  |  |  |  |
| 11 | 36383 | 3070 | 712 | 1261 | 30869 | 27986 | 2572 | 442 | 973 | 23593 | 27181 | 2426 | 422 | 933 | 23106 | 26052 | 2488 | 387 | 811 | 22092 |
| Overall | 195839 | 15012 | 5373 | 9480 | 163805 | 175451 | 14444 | 4809 | 8078 | 145567 | 182051 | 14404 | 4835 | 8699 | 152195 | 175379 | 15023 | 5284 | 7925 | 145391 |
| **Colorectal Cancer Screening** | | | | | | | | | | | | | | | | | | | | |
|  | **2019** | | | | | **2020** | | | | | **2021** | | | | | **2022** | | | | |
| Group | All | Asian | Black | Hispanic | White | All | Asian | Black | Hispanic | White | All | Asian | Black | Hispanic | White | All | Asian | Black | Hispanic | White |
| 1 | 22438 | 1290 | 766 | 651 | 19328 | 22953 | 1369 | 813 | 681 | 19603 | 24504 | 1647 | 982 | 873 | 20668 | 24495 | 1809 | 955 | 869 | 20508 |
| 2 | 3297 | 320 | 654 | 595 | 1686 | 3655 | 331 | 652 | 610 | 2007 | 3952 | 424 | 698 | 731 | 2058 | 3659 | 427 | 661 | 682 | 1852 |
| 3 | 6408 | 38 | 53 | 148 | 6158 | 6041 | 48 | 50 | 160 | 5774 | 5860 | 43 | 46 | 182 | 5579 | 11098 | 135 | 167 | 386 | 10395 |
| 4 | 46071 | 2324 | 800 | 1539 | 40896 | 46217 | 2592 | 799 | 1667 | 40463 | 45594 | 2967 | 872 | 1522 | 39800 | 43621 | 3061 | 807 | 1431 | 37953 |
| 5 | 54552 | 2041 | 713 | 1588 | 49532 | 77255 | 2730 | 968 | 2243 | 66154 | 63338 | 2710 | 804 | 2033 | 57274 | 62736 | 2927 | 787 | 1938 | 56583 |
| 6 | 7540 | 332 | 96 | 293 | 6750 | 8286 | 412 | 112 | 302 | 7364 | 8572 | 437 | 110 | 323 | 7664 | 8141 | 437 | 106 | 306 | 7271 |
| 7 | 8246 | 67 | 48 | 109 | 7831 | 8968 | 72 | 58 | 113 | 8474 | 9503 | 89 | 56 | 121 | 9031 | 8850 | 91 | 61 | 130 | 8386 |
| 8 | 4025 |  |  | 198 | 3781 | 3890 |  |  | 188 | 3658 | 3856 |  |  | 183 | 3622 | 3643 |  |  | 169 | 3419 |
| 9 | 21037 | 342 | 280 | 1038 | 19176 | 24702 | 490 | 502 | 1245 | 22254 | 35332 | 766 | 513 | 1737 | 32040 | 28862 | 734 | 440 | 1405 | 26107 |
| 10 | 1806 |  |  | 76 | 1678 | 1553 |  |  | 74 | 1442 | 1318 |  |  | 64 | 1223 | 1147 |  |  | 70 | 1034 |
| 11 | 24008 | 1486 | 410 | 696 | 21028 | 25040 | 1516 | 400 | 751 | 21954 | 26602 | 1638 | 418 | 787 | 23488 | 25249 | 1727 | 390 | 722 | 22159 |
| Overall | 199428 | 8240 | 3820 | 6931 | 177844 | 228560 | 9560 | 4354 | 8034 | 199147 | 228431 | 10721 | 4499 | 8556 | 202447 | 221501 | 11348 | 4374 | 8108 | 195667 |
| **Diabetes: BP control (<140/90 mm Hg)** | | | | | | | | | | | | | | | | | | | | |
|  | **2019** | | | | | **2020** | | | | | **2021** | | | | | **2022** | | | | |
| Group | All | Asian | Black | Hispanic | White | All | Asian | Black | Hispanic | White | All | Asian | Black | Hispanic | White | All | Asian | Black | Hispanic | White |
| 1 | 3016 | 326 | 270 | 173 | 2185 | 3196 | 348 | 304 | 189 | 2293 | 3242 | 358 | 330 | 203 | 2292 | 4020 | 449 | 534 | 292 | 2676 |
| 2 | 806 | 76 | 260 | 189 | 266 | 806 | 71 | 238 | 182 | 267 | 792 | 92 | 222 | 169 | 292 | 895 | 99 | 261 | 169 | 348 |
| 3 | 891 |  |  | 55 | 815 | 868 |  |  | 63 | 784 | 859 |  |  | 69 | 772 | 1200 |  |  | 94 | 1063 |
| 4 | 5745 | 446 | 244 | 385 | 4584 | 5622 | 440 | 224 | 359 | 4411 | 5246 | 459 | 218 | 315 | 4177 | 5599 | 523 | 261 | 329 | 4409 |
| 5 | 6439 | 398 | 219 | 431 | 5321 | 7889 | 492 | 243 | 526 | 6234 | 6900 | 478 | 211 | 518 | 5609 | 7422 | 542 | 297 | 543 | 5960 |
| 6 | 1174 | 88 |  | 73 | 970 | 1250 | 105 |  | 83 | 1014 | 1297 | 104 |  | 89 | 1049 | 1264 | 102 |  | 80 | 1026 |
| 7 | 673 |  |  |  | 608 | 673 |  |  |  | 592 | 1160 |  |  |  | 1053 |  |  |  |  |  |
| 8 | 754 |  |  | 52 | 690 | 754 |  |  | 52 | 667 | 682 |  |  | 47 | 625 |  |  |  |  |  |
| 9 | 3802 | 91 | 96 | 313 | 3259 | 3538 | 92 | 86 | 281 | 3036 | 5057 | 162 | 145 | 406 | 4287 | 4263 | 161 | 149 | 340 | 3573 |
| 10 | 321 |  |  |  | 282 | 285 |  |  |  | 250 | 247 |  |  |  | 211 |  |  |  |  |  |
| 11 | 3559 | 405 | 152 | 174 | 2775 | 3855 | 444 | 144 | 187 | 2944 | 3559 | 416 | 128 | 177 | 2792 | 3612 | 452 | 141 | 174 | 2802 |
| Overall | 27180 | 1830 | 1241 | 1845 | 21755 | 28736 | 1992 | 1239 | 1922 | 22492 | 29041 | 2069 | 1254 | 1993 | 23159 | 28275 | 2328 | 1643 | 2021 | 21857 |
| **Diabetes: Eye Exam** | | | | | | | | | | | | | | | | | | | | |
|  | **2019** | | | | | **2020** | | | | | **2021** | | | | | **2022** | | | | |
| Group | All | Asian | Black | Hispanic | White | All | Asian | Black | Hispanic | White | All | Asian | Black | Hispanic | White | All | Asian | Black | Hispanic | White |
| 1 | 3016 | 326 | 270 | 173 | 2185 | 3196 | 348 | 304 | 189 | 2293 | 3242 | 358 | 330 | 203 | 2292 | 4020 | 449 | 534 | 292 | 2676 |
| 2 | 806 | 76 | 260 | 189 | 266 | 827 | 89 | 233 | 189 | 300 | 792 | 92 | 222 | 169 | 292 | 895 | 99 | 261 | 169 | 348 |
| 3 | 891 |  |  | 55 | 815 | 870 |  |  | 63 | 786 | 859 |  |  | 69 | 772 | 1200 |  |  | 94 | 1063 |
| 4 | 5745 | 446 | 244 | 385 | 4584 | 5566 | 479 | 243 | 361 | 4407 | 5246 | 459 | 218 | 315 | 4177 | 5599 | 523 | 261 | 329 | 4409 |
| 5 | 6439 | 398 | 219 | 431 | 5321 | 7889 | 492 | 243 | 526 | 6234 | 6900 | 478 | 211 | 518 | 5609 | 7422 | 542 | 297 | 543 | 5960 |
| 6 | 1174 | 88 |  | 73 | 970 | 1250 | 105 |  | 83 | 1014 | 1297 | 104 |  | 89 | 1049 | 1264 | 102 |  | 80 | 1026 |
| 7 | 1101 |  |  |  | 1002 | 1143 |  |  |  | 1030 | 1160 |  |  |  | 1053 |  |  |  |  |  |
| 8 | 754 |  |  |  | 690 | 691 |  |  |  | 633 | 682 |  |  |  | 625 |  |  |  |  |  |
| 9 | 3802 | 91 | 96 | 313 | 3259 | 4425 | 128 | 180 | 367 | 3700 | 5057 | 162 | 145 | 406 | 4287 | 4263 | 161 | 149 | 340 | 3573 |
| 10 | 321 |  |  |  | 282 | 285 |  |  |  | 250 | 247 |  |  |  | 211 |  |  |  |  |  |
| 11 | 3559 | 405 | 152 | 174 | 2775 | 3523 | 409 | 131 | 175 | 2760 | 3559 | 416 | 128 | 177 | 2792 | 3612 | 452 | 141 | 174 | 2802 |
| Overall | 27608 | 1830 | 1241 | 1793 | 22149 | 29665 | 2050 | 1334 | 1953 | 23407 | 29041 | 2069 | 1254 | 1946 | 23159 | 28275 | 2328 | 1643 | 2021 | 21857 |
| **Diabetes: HbA1c poor control (>9.0%)** | | | | | | | | | | | | | | | | | | | | |
|  | **2019** | | | | | **2020** | | | | | **2021** | | | | | **2022** | | | | |
| Group | All | Asian | Black | Hispanic | White | All | Asian | Black | Hispanic | White | All | Asian | Black | Hispanic | White | All | Asian | Black | Hispanic | White |
| 1 | 3016 | 326 | 270 | 173 | 2185 | 3196 | 348 | 304 | 189 | 2293 | 3242 | 358 | 330 | 203 | 2292 | 4020 | 449 | 534 | 292 | 2676 |
| 2 | 806 | 76 | 260 | 189 | 266 | 834 | 77 | 244 | 186 | 292 | 792 | 92 | 222 | 169 | 292 | 895 | 99 | 261 | 169 | 348 |
| 3 | 891 |  |  | 55 | 815 | 868 |  |  | 63 | 784 | 859 |  |  | 69 | 772 | 1200 |  |  | 94 | 1063 |
| 4 | 5745 | 446 | 244 | 385 | 4584 | 5587 | 459 | 225 | 353 | 4401 | 5246 | 459 | 218 | 315 | 4177 | 5599 | 523 | 261 | 329 | 4409 |
| 5 | 6439 | 398 | 219 | 431 | 5321 | 6633 | 441 | 199 | 482 | 5433 | 6900 | 478 | 211 | 518 | 5609 | 7422 | 542 | 297 | 543 | 5960 |
| 6 | 1174 | 88 |  | 73 | 970 | 1250 | 105 |  | 83 | 1014 | 1297 | 104 |  | 89 | 1049 | 1264 | 102 |  | 80 | 1026 |
| 7 | 673 |  |  |  | 608 | 833 |  |  |  | 739 | 1160 |  |  |  | 1053 |  |  |  |  |  |
| 8 | 754 |  |  |  | 690 | 691 |  |  |  | 633 | 682 |  |  |  | 625 |  |  |  |  |  |
| 9 | 3802 | 91 | 96 | 313 | 3259 | 3538 | 92 | 86 | 281 | 3036 | 5057 | 162 | 145 | 406 | 4287 | 4263 | 161 | 149 | 340 | 3573 |
| 10 | 321 |  |  |  | 282 | 285 |  |  |  | 250 | 247 |  |  |  | 211 |  |  |  |  |  |
| 11 | 3559 | 405 | 152 | 174 | 2775 | 3855 | 444 | 144 | 187 | 2944 | 3559 | 416 | 128 | 177 | 2792 | 3612 | 452 | 141 | 174 | 2802 |
| Overall | 27180 | 1830 | 1241 | 1793 | 21755 | 27570 | 1966 | 1202 | 1824 | 21819 | 29041 | 2069 | 1254 | 1946 | 23159 | 28275 | 2328 | 1643 | 2021 | 21857 |
| **Controlling High Blood Pressure** | | | | | | | | | | | | | | | | | | | | |
|  | **2019** | | | | | **2020** | | | | | **2021** | | | | | **2022** | | | | |
| Group | All | Asian | Black | Hispanic | White | All | Asian | Black | Hispanic | White | All | Asian | Black | Hispanic | White | All | Asian | Black | Hispanic | White |
| 1 | 9538 | 648 | 571 | 360 | 7760 | 9143 | 595 | 590 | 347 | 7356 | 6726 | 468 | 507 | 298 | 5349 | 8736 | 665 | 778 | 388 | 6783 |
| 2 | 2146 | 177 | 598 | 405 | 938 | 2095 | 166 | 518 | 362 | 941 | 1766 | 176 | 414 | 343 | 813 | 1911 | 182 | 493 | 332 | 881 |
| 3 | 2872 |  | 46 | 110 | 2694 | 2615 |  | 40 | 106 | 2444 | 2067 |  | 30 | 89 | 1936 | 4447 |  | 98 | 208 | 4092 |
| 4 | 19598 | 1136 | 609 | 972 | 16628 | 19586 | 1049 | 587 | 899 | 15865 | 13592 | 917 | 454 | 586 | 11511 | 14476 | 1078 | 512 | 612 | 12156 |
| 5 | 23162 | 961 | 533 | 978 | 20435 | 34619 | 1316 | 755 | 1406 | 28813 | 19975 | 847 | 440 | 892 | 17642 | 21253 | 1020 | 511 | 889 | 18646 |
| 6 | 3361 | 160 | 72 | 173 | 2919 | 3642 | 213 | 81 | 181 | 3110 | 2759 | 150 | 65 | 151 | 2381 | 2678 | 148 | 64 | 125 | 2333 |
| 7 | 2639 |  |  |  | 2474 | 2639 |  |  |  | 2382 | 3307 |  |  |  | 3111 | 3596 |  |  |  | 3359 |
| 8 | 2470 |  |  | 137 | 2303 | 2470 |  |  | 135 | 2248 | 1835 |  |  | 94 | 1721 | 1801 |  |  | 94 | 1677 |
| 9 | 11556 | 171 | 223 | 669 | 10366 | 10682 | 175 | 210 | 608 | 9569 | 15589 | 334 | 348 | 933 | 13833 | 12896 | 320 | 315 | 746 | 11424 |
| 10 | 935 |  |  | 73 | 823 | 810 |  |  | 60 | 723 | 626 |  |  | 49 | 557 | 550 |  |  | 54 | 474 |
| 11 | 12373 | 854 | 345 | 461 | 10509 | 13112 | 841 | 322 | 502 | 10786 | 9843 | 717 | 244 | 364 | 8415 | 9733 | 782 | 286 | 361 | 8194 |
| Overall | 90650 | 4107 | 2997 | 4338 | 77849 | 1E+05 | 4355 | 3103 | 4606 | 84237 | 78085 | 3609 | 2502 | 3799 | 67269 | 82077 | 4195 | 3057 | 3809 | 70019 |

Note: Empty cells are due to redacted observations that were less than the minimum 50 average per year for the racial and ethnic patient groups examined.
